# Continuous Attractor Network Model for Conjunctive Position-by-Velocity Tuning of Grid Cells

**DOI:** 10.1371/journal.pcbi.1003558

**Published:** 2014-04-17

**Authors:** Bailu Si, Sandro Romani, Misha Tsodyks

**Affiliations:** 1 Department of Neurobiology, Weizmann Institute of Science, Rehovot, Israel; 2 Center for Theoretical Neuroscience, Columbia University, New York, New York, United States of America; The University of Chicago, United States of America

## Abstract

The spatial responses of many of the cells recorded in layer II of rodent medial entorhinal cortex (MEC) show a triangular grid pattern, which appears to provide an accurate population code for animal spatial position. In layer III, V and VI of the rat MEC, grid cells are also selective to head-direction and are modulated by the speed of the animal. Several putative mechanisms of grid-like maps were proposed, including attractor network dynamics, interactions with theta oscillations or single-unit mechanisms such as firing rate adaptation. In this paper, we present a new attractor network model that accounts for the conjunctive position-by-velocity selectivity of grid cells. Our network model is able to perform robust path integration even when the recurrent connections are subject to random perturbations.

## Author Summary

How do animals self-localize when they explore the environments with variable velocities? One mechanism is dead reckoning or path-integration. Recent experiments on rodents show that such computation may be performed by grid cells in medial entorhinal cortex. Each grid cell fires strongly when the animal enters locations that define the vertices of a triangular grid. Some of the grid cells show grid firing patterns only when the animal runs along particular directions. Here, we propose that grid cells collectively represent arbitrary conjunctions of positions and movements of the animal. Due to asymmetric recurrent connections, the network has grid patterns as states that are able to move intrinsically with all possible directions and speeds. A velocity-tuned input will activate a subset of the population that prefers similar movements, and the pattern in the network moves with a velocity proportional to the movement of the animal in physical space, up to a fixed rotation. Thus the network ‘imagines’ the movement of the animal, and produces single cell grid firing responses in space with different degree of head-direction selectivity. We propose testable predictions for new experiments to verify our model.

## Introduction

Responses of grid cells recorded in medial entorhinal cortex (MEC) provide accurate population codes for the positions in an environment [1], and could result from path-integration mechanism [2]. Attractor network models of MEC spatial representations have been proposed, based on two foundations. First, they assume surround-inhibition recurrent connections, such as in Mexican-hat type connection profile, between grid cells [3]–[7]. When sufficiently strong, surround-inhibition connections endow recurrent networks with stationary hexagonal patterns of activity patches even when driven by uniform external inputs [3], [5], [8]. In the absence of external cues, this pattern can have an arbitrary spatial phase and orientation, a hallmark of a *continuous attractor model*. In order to generate individual cells with an hexagonal pattern of firing fields from an hexagonal pattern of network activity, the latter has to flow across the network with a velocity vector proportional to the velocity of the animal, up to a fixed rotation. In [5], this movement is emerging in the network via a combination of two additional mechanisms: (i) each neuron receives a speed-modulated input that is tuned for a particular direction of movement; and (ii) the neuron's outgoing connections are slightly shifted in the direction reflecting the neuron's preferred direction. As a result, when the animal is moving in a certain direction, the neurons that prefer this direction are slightly more active than their counterparts, and generate the appropriate flow across the network sheet. While the model of [5] was shown to generate robust grid cells, it cannot account for cells that are strongly directionally tuned (“conjunctive cells”) [9]. Moreover, in order to produce stable firing fields, the flow speed should be precisely proportional to the animal velocity, which can only be achieved by the abovementioned mechanism with threshold-linear neurons, not a realistic assumption given strong nonlinearities of neuronal firing mechanism.

In order to develop a robust continuous attractor model of grid system, we suggest that MEC networks contain intrinsic representations of arbitrary *conjunctions* of positions and movements of the animal. To achieve such representations, we construct a network with grid-like activity patterns that are intrinsically moving with different velocities, as opposed to stationary patterns in the earlier models. Individual neurons in the network are labeled with different position/velocity combinations, and connectivity is configured in such a way that activity bumps, when centered on neurons with particular velocity labels, are intrinsically moving at the corresponding speed and direction. The appropriate positioning of the activity bumps is assumed to be achieved by the velocity-dependent input as in [5]. The mapping between the animal movement and the position of the bumps on the velocity axis can be learned by the network during development, such that the velocity of the bumps in the neural space is proportional to the velocity of the animal in the physical space. The network thus performs path-integration and forms stable grid maps in the environment. We demonstrate that this model does not require precise tuning of recurrent connections and naturally accounts for the co-existence of pure grid cells and strongly directional, conjunctive cells.

## Results

Each unit in the network is assigned a set of coordinates on a manifold embedded in a patch of MEC. The dimensions of the neuronal manifold represent the position or velocity of the animal in its environment. The activity of the units is governed by the dynamics of the interactions between units conveyed by recurrent connections. To illustrate the basic ideas of the model, we first consider a one-dimensional environment, i.e. a linear track on which the animal runs back and forth. The results for two dimensional environments are presented in *Results - Two dimensional environment*.

### Neural representation of position and velocity

On a linear track, the network only needs to encode one-dimensional locations and integrate one-dimensional velocity. Each unit is labeled by its coordinates 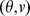 on an abstract 2-dimensional neuronal manifold. 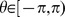 is an internal representation of the positions of the environment. For simplicity we assume periodic boundary conditions in 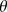. Note that the physical environment has fixed boundary conditions, and the simulated animal can not go beyond the boundaries. Both 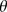 and 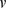 are dimensionless quantities, but they reflect physical position and velocity of the animal (see below). We choose the connections between the units such that the network has multiple bumps in the position dimension and a single bump in the velocity dimension. In this paper we consider the simplest choice for the connections from unit 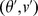 to unit 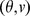 in the following form (but we expect the precise form not to be important for the qualitative behavior of the network):
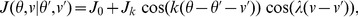
(1)where 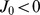 is uniform inhibition, 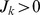 defines the range of interaction strengths, 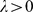 is the strength of velocity tuning and 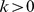 is an integer, determining the number of bumps in the dimension of 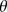. Note that for each value of 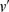 connections are asymmetric in the position dimension, which results in the bumps moving along this dimension with the speed and direction determined by 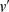. Throughout the paper we choose 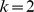 and 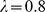. The former choice was taken in order to be consistent with the 2D case described below, where several activity bumps are required for the neurons to exhibit triangular grid fields, without endowing the abstract neural tissue with twisted torus boundary conditions necessary for previous models [10], [11]. The range of 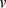 is chosen to be 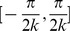, since for values of 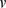 beyond this range the moving bump solution disappears (see Eq. 14 and *Methods* *- Speed estimation in the asymmetric ring model*).

**Figure 1:**
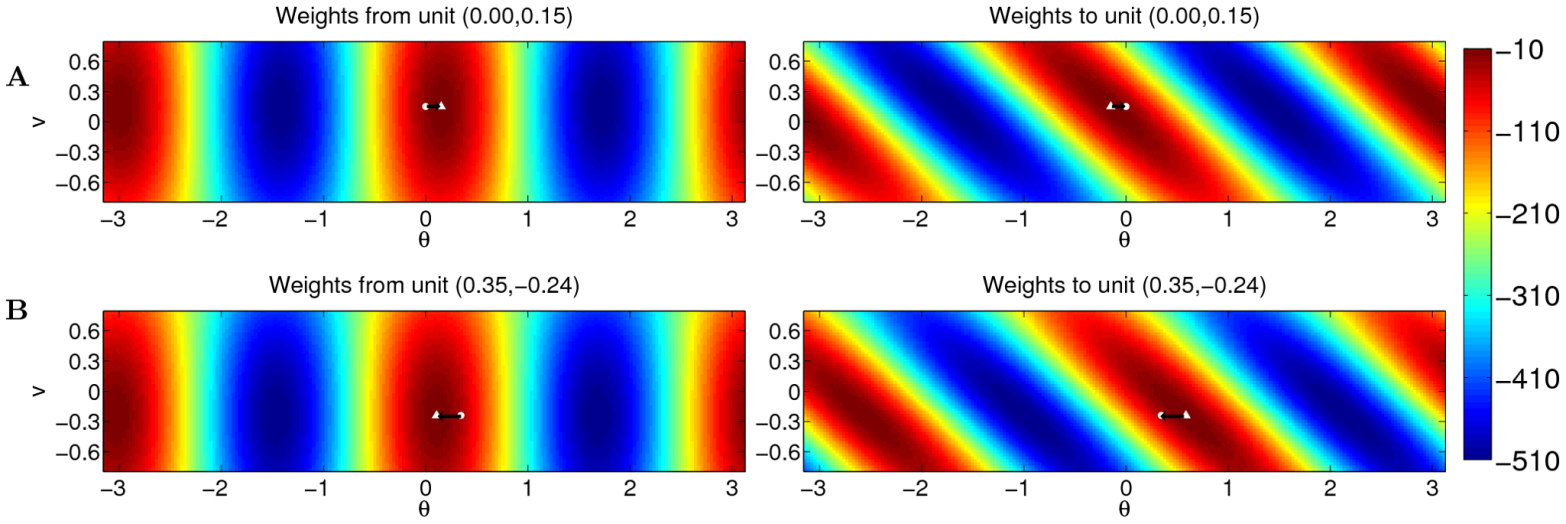
Weights of two example units on a neural manifold of position and velocity (parameters: 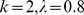). The weight profile has 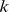 periods along the spatial dimension. (A) The outgoing weight (left panel) and the incoming weight (right panel) of the unit at 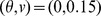 (marked by white dots). The outgoing weight profile of the unit is not centered at its own location in the position dimension, but rotated 0.15 radians to the right (white triangle). The amount of the shift is determined by the velocity label of the unit, as indicated by the black arrow. In the velocity dimension, the connections show broad modulation (the peak of the weight profile marked by the white triangle). The incoming weights (right panel) to the same unit (white circle) is tilted, since the unit receives strong connections from units in the left/right with a positive/negative shift determined by the projection units, among which the maximal activation comes from the unit 0.15 to the left (marked by white triangle) due to the modulation in the velocity dimension; (B) The outgoing weight (left panel) and the incoming weight (right panel) of the unit with negative velocity label, 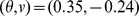 (marked by white dots). The outgoing weight profile is centered (white triangle) to the left of the unit in the spatial dimension, due to the negative velocity label of the unit. The incoming weight of the unit is tilted, with the maximal connection coming from the right.

The outgoing weight profile of a unit is not centered at its own spatial label, but is shifted by an amount determined by its velocity label (Fig. 1A). The weight profile is broadly modulated in the velocity dimension by the second cosine term of Eq. 1. The incoming weights of a unit is shifted in the spatial axis by amounts determined by presynaptic units, showing tilted patterns (Fig. 1B), a structure imposed by the term 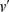 in the first cosine term of Eq. 1.

### Intrinsic network dynamics

We first consider the intrinsic activity of the network without a velocity tuned input. The firing rate of the unit at 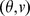 is denoted by 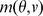. The dynamics of the network activity is described by
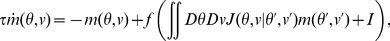
(2)where 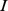 is a uniform input current, 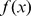 is a transfer function typically defined as a threshold-linear function if not stated explicitly: 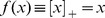 when 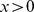 and 0 otherwise. The notations 
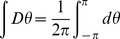
, and 
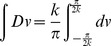
.

The coupling can be rewritten as
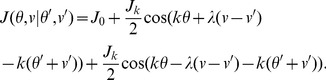
(3)This model is mathematically similar to the model discussed in [12], but with 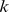 bumps and asymmetrical connections in 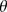.

### Order parameters

The properties of the network activity 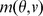 can be characterized by an appropriately chosen set of order parameters. Thanks to the ring connectivity structure used, we introduce five order parameters (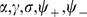) to describe the network activity [12], [13]. The dynamics of the firing rate can be rewritten in terms of these order parameters as (see *Methods* *- Order parameters* for the detailed derivation)
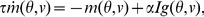
(4)where 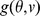, the rescaled input (see Eq.27 below), is defined by
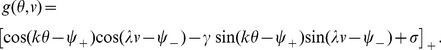
(5)


The dynamics of the order parameters are governed by following equations:
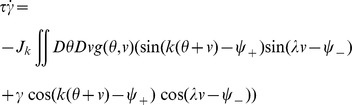


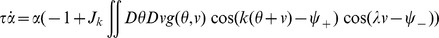


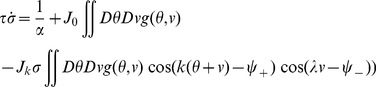
(6)

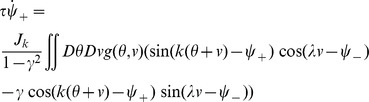


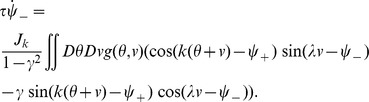




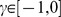
 defines the slant of the bumps, 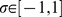 is the threshold that sets the size of the bumps, 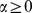 is the amplitude of the bumps in the network. 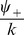 and 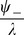 indicate the peak location of the bumps in 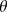 and 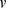 dimensions respectively.

### Solutions

**Figure 2:**
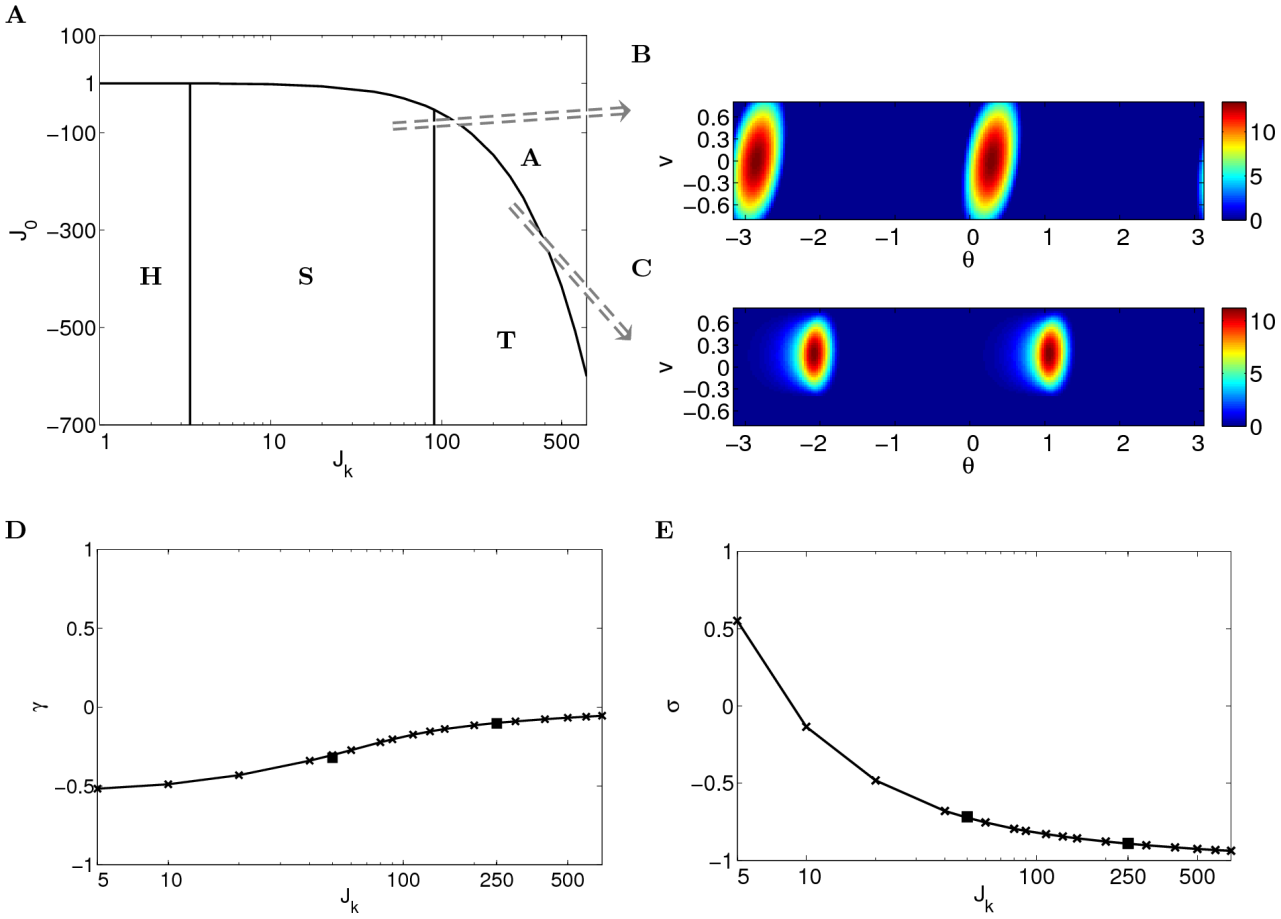
Depending on the parameters, the network operates in different regimes. (A) The amplitude instability (A) is separated from the homogeneous regime (H) and localized activity regimes (S and T) by Eq. 7 and 12. Localized regimes are separated from the homogeneous regime by Eq. 8. The regime of traveling bumps (T) is separated from the regime of static bumps (S) by Eq. 35; (B) An example of the network state in localized activity regime (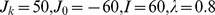). (C) An example of traveling bumps (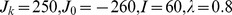); (D–E) Fixed point solutions of order parameters 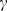 and 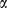 for various 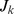. The square markers correspond to the order parameters of the examples shown in (A). With larger 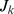, the bumps in the network are less tilted (larger 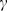) and smaller (smaller 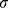).

The solutions to the system in Eq. 2 show qualitatively different forms depending on the parameters 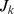 and 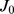. If 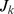 is small, the network activity is uniform (homogeneous regime, Fig. 2A). When 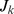 increases, the network activity converges to 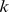 bumps, localized at the arbitrary stationary position 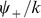 in 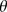 dimension and spanning the whole range in 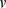 dimension (static bumps regime; 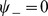, see Fig. 2B). The forces from the units with positive (negative) velocity labels in propagating the bumps to right (left) balance each other, therefore the bumps are static.

For sufficiently large 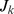, the bumps become localized also in the velocity dimension at the position 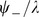 (Fig. 2C). Due to the asymmetry of the coupling in the spatial axis, the bumps start to move intrinsically along the spatial axis with velocity dependent on their position on the velocity axis (traveling bumps regime). Since the network forms a continuous attractor manifold in 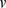 dimension, the bumps are free to be stabilized in the velocity axis and are able to move with a range of possible velocities along the spatial axis. In the traveling bumps regime, the network activity 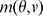 does not have any steady state, but the order parameters 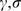 and 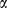 converge to fixed points. 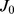 should be sufficiently negative in order to keep the network activity from explosion (amplitude instability regime). Throughout the paper, we assume inhibitory connections (i.e. 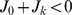) for convenience, although using excitatory connections will lead to similar results.

In this section, we analyze the fixed point solutions to the dynamics of the order parameters, and perform simulations to confirm the solutions found. Before analyzing the moving bumps regime, we briefly mention the homogeneous regime and static bumps regime for the sake of completeness.

#### Homogeneous solution

A trivial solution of the firing rate dynamics is a uniform activity in the network. We directly analyze the steady state of the system in Eq. 2. The steady state is 
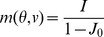
, imposing the condition
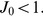
(7)The line separating the homogeneous regime from the static bumps regime is
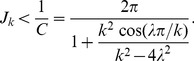
(8)where 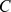 is defined as
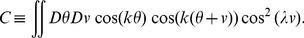
(9)The derivation of this result is detailed in *Methods* *- Stability of a homogeneous solution*.

#### Stationary activity bumps

When 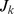 goes beyond 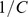, stationary bumps emerge in the network, that span the whole range of 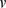
's. In this case, 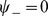, and 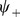 is a free parameter, defining the spatial position of the bumps. In the following, we choose 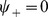. At the steady state, the network activity takes the form
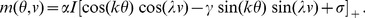
(10)


One example of static bumps is shown in Fig. 2B, simulated according to the rate dynamics in Eq. 2 (ref. *Methods* *- Network simulations* for details of simulations). The 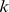 bumps in the network are tilted, corresponding to negative 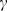 (Ref. *Methods* *- Order parameters*). The degree of the slant is proportional to the absolute value of 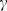.

The first three order parameters 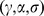 in Eqs. 6 converge to fixed point solutions:
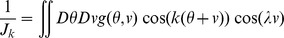


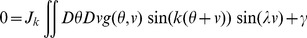
(11)

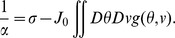
Here 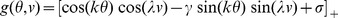.

From the last Eq. in 11, the condition for 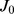 to avoid amplitude instability is
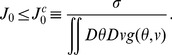
(12)


The fixed point equations 11 are not easy to deal with analytically. Instead, we resort to numerical integration to calculate the fixed-point solutions. Since the first two of the Eqs. 11 are decoupled from 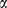, we solve 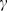 and 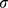 from the first two equations and then determine the range of 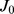 according to Eq. 12. Fig. 2D–E shows the fixed point solutions of 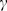 and 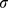. The critical value 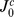 is plotted in Fig. 2A. The order parameters of the simulations in Fig. 2B–C match the numerical solutions (square markers in Fig. 2D–E).

#### Traveling bumps

The regime we are most interested in is the one in which multiple traveling bumps exist in the neural space. This requires the size of the bumps to be sufficiently small so that they can move freely along the 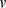 axis. The condition for this is 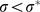, where (see *Methods* *- Onset of traveling bumps*)
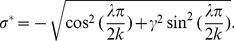
(13)Comparing Eq. 13 with the fixed point solution of 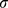 from the first two of Eqs. 11 allows defining the 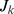 above which nonzero 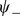 emerges and the bumps start to move. Fig. 2C shows one example of traveling bumps.

The velocity of the traveling bumps is given by 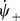 in Eqs. 6. It depends on the center 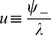 of the bumps on velocity axis. Although a closed form of the functional relation between 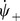 and 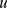 is not tractable, it can be approximated by the velocity of the bumps when the manifold is reduced to a ring defined on 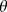 for a fixed 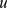 (see *Methods* *- Speed estimation in the asymmetric ring model*)
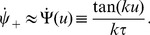
(14)


**Figure 3:**
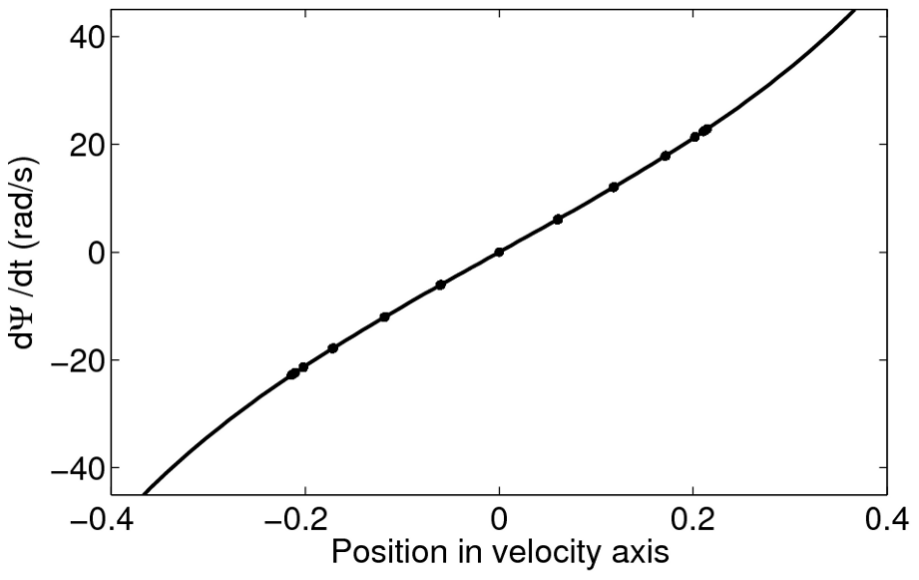
The instantaneous velocity of the traveling bumps is well described by the approximation 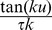 (solid curve). The bumps are put at 11 different positions on the velocity axis. Each circle shows the instantaneous velocity of the bumps during a 1 ms step in the one-second simulation. Overlapping circles demonstrate stable intrinsic velocity of the bumps. For the parameters used, the bumps cannot be put to positions 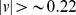 on the velocity axis since the bumps touch the border 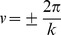.

We calculate the intrinsic velocity of the bumps by simulating the network with uniform input (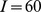, ref. *Methods* *- Estimating the intrinsic velocity of the bumps*). Indeed, the instantaneous velocity of the bumps, as a function of the center of the bump positions on the velocity axis, fits well with the approximation (solid curve in Fig. 3) given in Eq. 14. The bumps are stable in the velocity axis (see Fig. 3).

### Velocity tuned input

In order to perform path-integration, or equivalently to form stable firing maps, the velocity of the bumps has to be kept proportional to the velocity of the animal
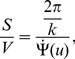
(15)where 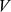 is the velocity of the animal, 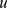 is the desired position on the velocity axis, 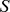, the spacing between grid fields, is a scaling factor between the velocity of the animal in physical space and the velocity of the bumps in neural space. Eq. 15 means that for any velocity of the animal the time it takes for the animal to travel in physical space between two grid fields is equal to the time it takes for the bumps in neural space to flow for one period with desired velocity 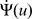. For a given 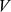, Eq. 14, 15 can be solved for
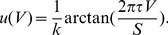
(16)The function 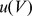 therefore tells where the bumps should be located in 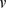 dimension given the velocity 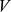 of the animal, i.e. where the velocity tuned external input should be pointing. We choose the velocity-tuned input to the network given by Gaussian tuning
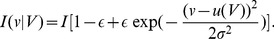
(17)Here 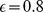 is the strength of the velocity tuning, 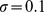 is the sharpness of the tuning, 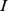 is the amplitude of the input as before. Note that in the brain the function 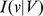 can be implemented by a neural network, the connections of which may be learned during development of the MEC. For simplicity, we assume in this paper that such a network has already been formed during appropriate developmental stages [14]–[16]. In all the simulations presented in this paper, we only consider inputs that are untuned in the spatial dimension, in order to study the ability of the network to perform path integration in the absence of sensory cues. Adding such cues will make the grid fields more robust.

#### Path integration on linear track

**Figure 4:**
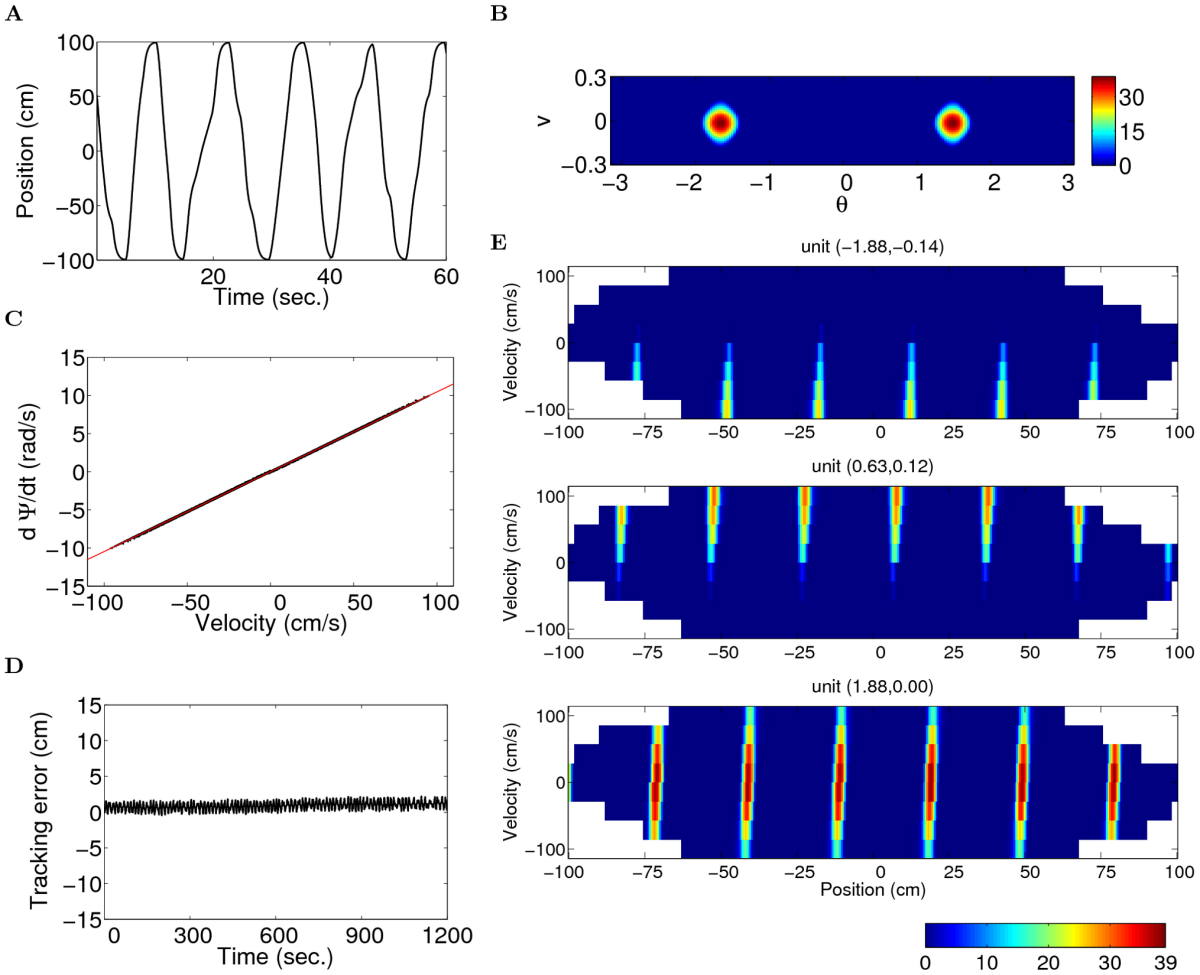
The units develop stable position-by-velocity maps on a two-meter linear track in a simulation of 20 minutes (parameters: 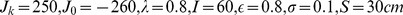). (A) Part of the trajectory of the virtual animal. (B) One snapshot of the network activity during the simulation. (C) The velocity of the bumps is linearly related to the velocity of the virtual rat. Every 100 ms, the instantaneous velocities of the bumps and the animal during 1 ms interval is shown by a dot in the plot. The line shows the slope 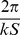, ref. Eq. 15; (D) The tracking error (the difference between the estimated position and the actual position of the animal) is small compared to the spacing (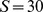 cm). (E) Position-by-velocity maps of two conjunctive units (top two rows) and a grid unit (bottom). The coordinate 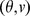 in the neural space is indicated at the top of each panel. Non-sampled bins are represented by white color.

We simulated an animal running back and forth on a two-meter-long linear track. The velocity of the virtual animal is simulated according to a continuous random walk with the constraint that the animal can not leave the boundaries of the track and that the peak speed is 100 cm/s (ref. *Methods* *- Network simulations* and Video S1). Fig. 4A shows one minute trajectory from the simulation. Due to velocity-tuned input, the size as well as the slant of the bumps in the network is reduced (Fig. 4B vs. 2C). The bumps are shifted along the velocity axis by the velocity-tuned input to desired locations. As a result, the velocity of the bumps is approximately proportional to the velocity of the animal (Fig. 4C). The slope of the linear relation is 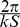 as given by Eq. 15. We estimate the position of the animal by considering the phase history of the bumps. The difference between the estimated and the actual position of the animal is bounded during the whole simulation (Fig. 4D), demonstrating accurate path-integration in the network.

The position-by-velocity maps of three example units on the linear track are shown in Fig. 4E. In the 20-minute simulation, all units develop stable fields. The spacing between the fields in the spatial dimension is 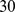 cm, as dictated by the parameter 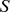 in the simulation. Depending on their position on the velocity axis of the neural space, units respond to different range of movements of the virtual rat. For example, units shown in the first two rows of Fig. 4E are only active when the animal runs along one direction, since these two units prefer high speed in one direction. In contrast, the unit shown below is not directional, because its velocity label is close to zero on the velocity axis, therefore it is active in both directions.

The center of the spatial fields is shifted towards the running direction (Fig. 4E). This is due to the slant of the bumps in the network (negative 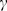). Each unit will be active when the bumps are placed more upper right or lower left relative to the unit. In the simulation shown in Fig. 4B, the slant of the bumps is rather weak due to strong input tuning, resulting in a weak shift of the spatial fields. If however, the velocity input tuning were reduced (smaller 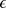), this *slant effect* of the fields would be stronger, since the shape of the bumps would be more similar to the case of uniform input.

The firing rates of conjunctive units are smaller than the firing rates of grid cells, as can be seen from the peak rates of the units in Fig. 4E. This is consistent with the analysis of the bump amplitude (see Eq. 57).

#### Robustness of the network

The wiring of the neural circuits of the brain can be irregular and imprecise. It was shown that continuous attractor networks are structurally unstable to perturbations in recurrent connections, which break the symmetry of the model and result in small number of discrete attractors [17]. Here we show that since we consider the moving activity bumps resulting from asymmetric connections, the network is robust to such perturbations in the recurrent weights.

**Figure 5:**
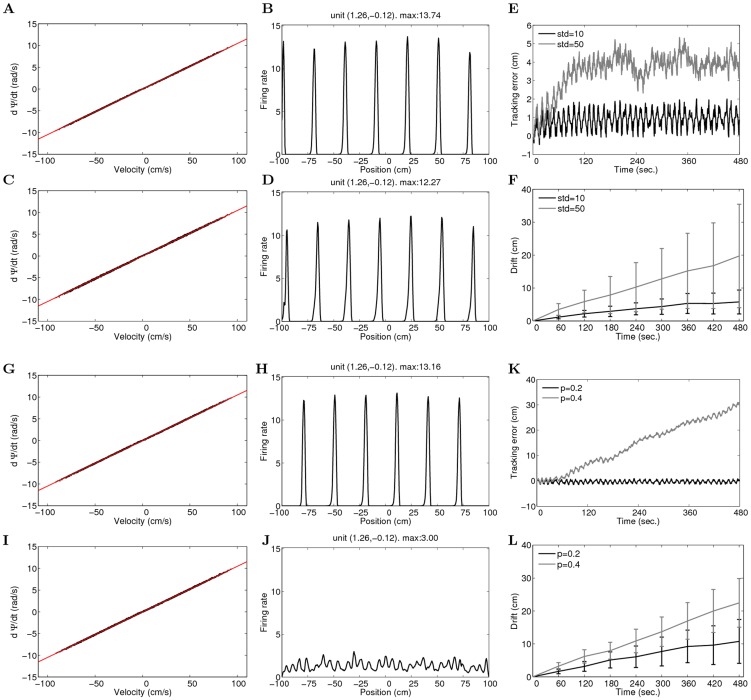
The network performs robust path-integration against perturbations in weights (parameters: 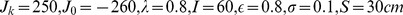). (A–F) Perturbation by Gaussian random noise with zero mean and standard deviation 2% or 10% relative to the weight range. A,C: Scatter plots of the velocity of the bumps with respect to the velocity of the virtual animal for 2% perturbation (A) or 10% perturbation (C). Every 100 ms in the simulation, the instantaneous velocities of the bumps and the animal during 1 ms interval is marked by a dot. The line indicates the slope 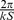 derived from Eq. 15; B,D: Spatial fields of two example units in the network with 2% (B) or 10% Gaussian perturbation (D); E: Tracking error, i.e. the difference between the estimated position from the network activity and the actual position of the animal; F: Drift, defined as the absolute value of tracking error, averaged across eight independent simulations. (G–L) Dilution of connectivity by 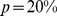 or 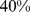. The weights are rescaled by 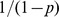 after the dilution to keep the strength of the connections comparable to the original connections. G,I: The relation between the velocity of bumps and the velocity of the animal. The same legends are used as in A; H,J: Spatial fields of two example units from the network with 20% (H) or 40% dilution; K: Tracking error; L: Drift.

We add to the entries of the weight matrix random numbers sampled from Gaussian distributions with zero mean and standard deviation equal to 2% or 10% of the range of the original weights (i.e. 10 or 50 for 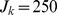). After Gaussian perturbations, the velocity of the bumps is kept roughly linear with respect to the velocity of the animal (Fig. 5A for 2% and C for 10% perturbation), showing small dispersions. In both simulations, units form stable grid field (Fig. 5B and D). The tracking errors are limited to up to 16% relative to the spacing (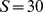 cm), although in the simulations with 10% perturbation the error shows larger fluctuations (Fig. 5E). We quantify the performance of path-integration by averaging drift, i.e. the absolute tracking error, across eight independent simulations with different random number sequence. With 10% Gaussian perturbation, the network is able to path-integrate for about five minutes before the drift reaches half of the spacing (Fig. 5F). For smaller perturbation, the network is able to path integrate for longer time.

We dilute the wiring of the network by randomly setting 20% or 40% of the elements in the weight matrix to zeros. In both simulations, the velocity of the bumps varies approximately in a linear fashion with respect to the velocity of the animal (Fig. 5G,I). For 20% dilution, the units in the network form sharp fields (Fig. 5H), and the tracking error of the network is small. In the simulation with 40% dilution of connections, however, units do not show clear firing field on the track (Fig. 5J). This is because tracking errors can accumulate over time, due to the lack of exact linear relationship between the velocity of the bumps and the velocity of the animal. After three minutes the network looses track of the position of the animal (Fig. 5K). When averaged across eight trials, the network is able to path-integrate for about five minutes with 40% dilution in connections (Fig. 5L).

The robustness of the velocity of the bumps in the network comes from the fact that it is intrinsically determined by the asymmetry of the connections and does not depend on the amplitude of the movement input. Moreover, the network forms continuous attractor manifold in the velocity dimension, allowing the bumps pinned to the desired position on the velocity axis to let the bumps travel with the appropriate velocity.

#### Nonlinear network

The firing rate of a neuron in the brain can be a highly nonlinear function of the afferent input. A more general transfer function of the model in Eq. 2 would be the threshold-sigmoid
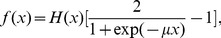
(18)where 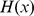 is the Heaviside function and 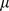 is the gain of the transfer function. The maximal firing rate is normalized to be 1.

**Figure 6:**
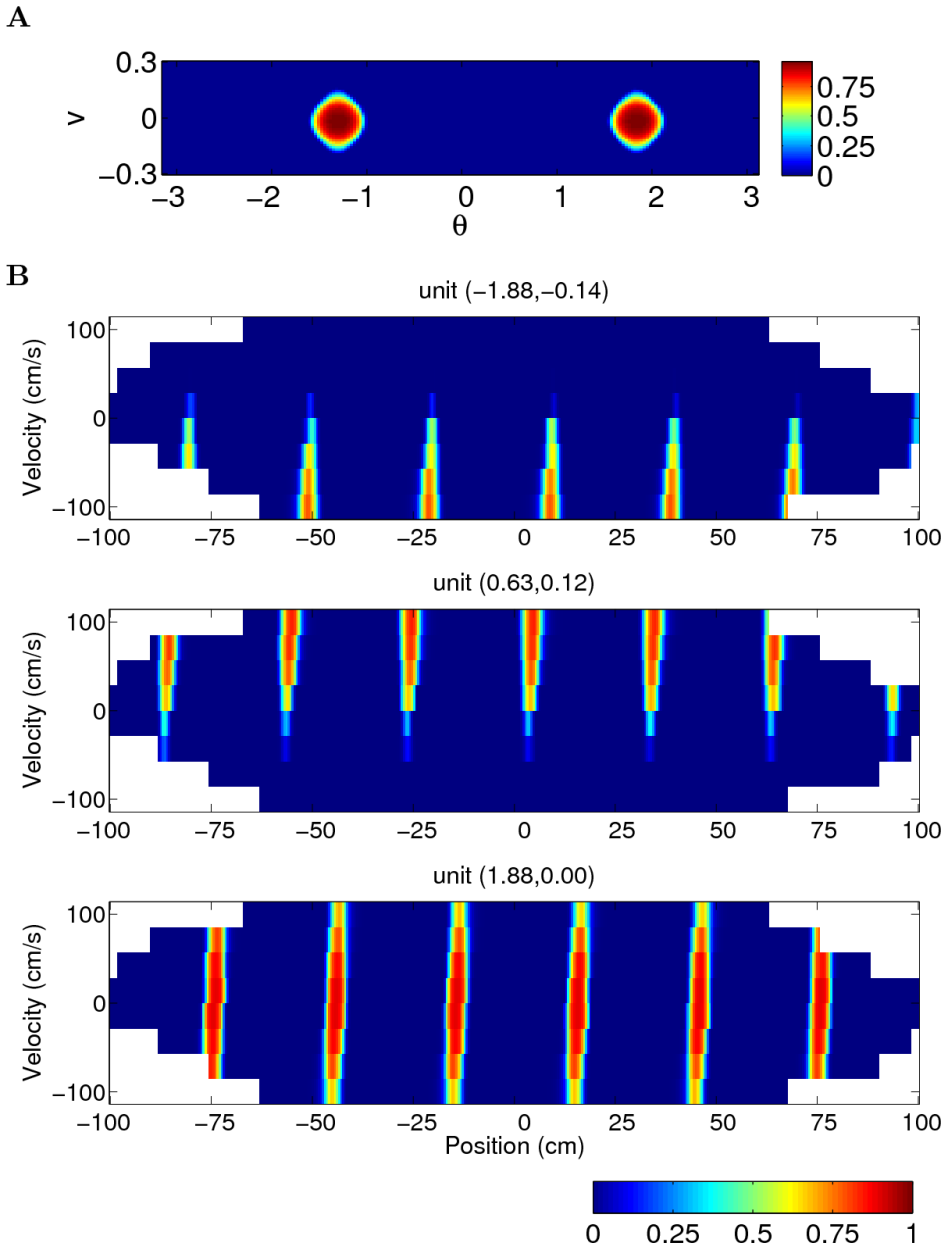
The network is able to perform accurate path-integration even when the firing response is nonlinear in the input and the velocity input is of finite resolution (parameters: 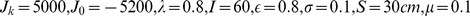). (A) Snapshot of the network activity at one example step in the simulation. The firing of the units in the network saturates due to nonlinearity of the transfer function; (B) Firing maps of the units, as a function of the actual position and velocity of the simulated rat, show that the top two units are conjunctive grid units while the unit at the bottom is a pure positional grid unit. The coordinate 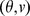 in the neural space is indicated at the top of each panel. The spacing is 30 cm, determined by the parameter 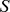 put in the simulation. Non-sampled bins are represented by white color.

In general, an analytical form of the mapping between the animal movement and the position of the bumps on the velocity axis as Eq. 16 is intractable. In the model, the mapping can be realized by a neural network that connects movement-selective units to the units of the model. For simplicity we use a lookup table, which has 201 bins of equal width in the range 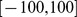 cm/s, as a substitute of the mapping. We compute the velocity of the bumps as a function of the position on the velocity axis from simulation. It turns out that the velocity of the bumps matches Eq. 16 for different 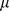 even when 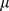 goes to infinity (i.e. binary units). We build the lookup table containing for each velocity bin the mapped position on the velocity axis calculated according to Eq. 16, using the center of the corresponding velocity bin as the argument. To compensate for the effect of the normalized activity, we scale up the strength of the connections by 20 times, so that the size of the bumps is similar to the linear case (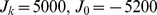, Fig. 6A).

We feed such a nonlinear network with velocity-tuned input given as in Eq. 17 but with 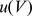 replaced by the lookup table. The units in the network still express stable grid fields during the 20 minutes simulation (Fig. 6B), meaning that the network is able to perform accurate path-integration even when the firing response is nonlinear in the input and the velocity input is of finite resolution.

### Two dimensional environment

In a high-dimensional environment, the neural space is expanded to represent position and velocity in each dimension of the physical space. For a two-dimensional environment, units in the neural space are labeled by coordinates 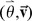. 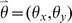 and 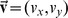 jointly represent the four-dimensional space of position and velocity in a two dimensional environment. For mathematical convenience, 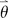 is assumed to have periodic boundary conditions, i.e. 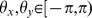. 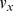 and 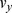 are in the range of 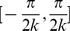.

**Figure 7:**
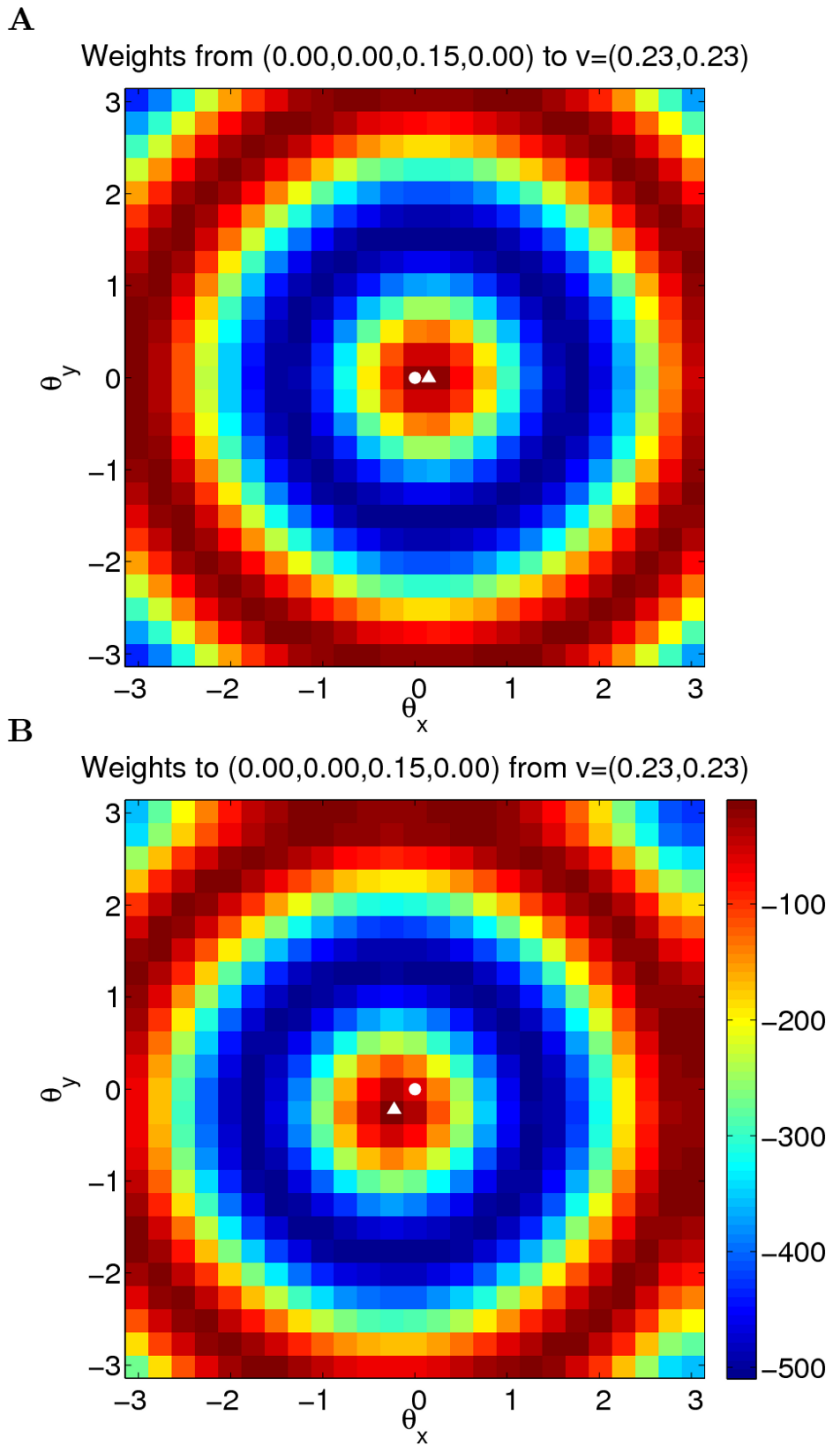
Weight matrix in four dimensional neural space 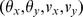. Only the slices at 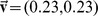 of the outgoing weights (A) from and incoming weights (B) to the example unit 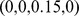 are shown. (A) The asymmetry in the outgoing weights is determined by the projecting unit (white dot). The triangle marks the unit that is maximally activated among the units in the slice by the projecting unit. (B) The asymmetry in the incoming weights depends on the velocity labels of presynaptic units. Among the unit in the slice, the unit marked by the triangle has the strongest connection to the example unit (white dot).

The weight matrix between units is defined as an extension of the one-dimensional case
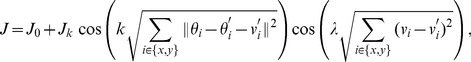
(19)where 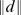 is the distance on a circle
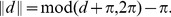
(20)Here mod(x,y) 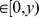 gives x modulo y. As can be seen from Eq. 19, the velocity label 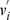 of the presynaptic units in the first cosinus term introduces an asymmetry to the weight matrix in the spatial axis. The second cosinus term is responsible for velocity selectivity. We simulate a network with units uniformly arranged in 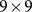 velocity bins and 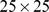 spatial bins in the neural space, summing up to 50,625 units in total in the network. Fig. 7 shows the weights between one example unit at 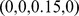 and units with velocity label 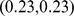 on the neural tissue.

**Figure 8:**
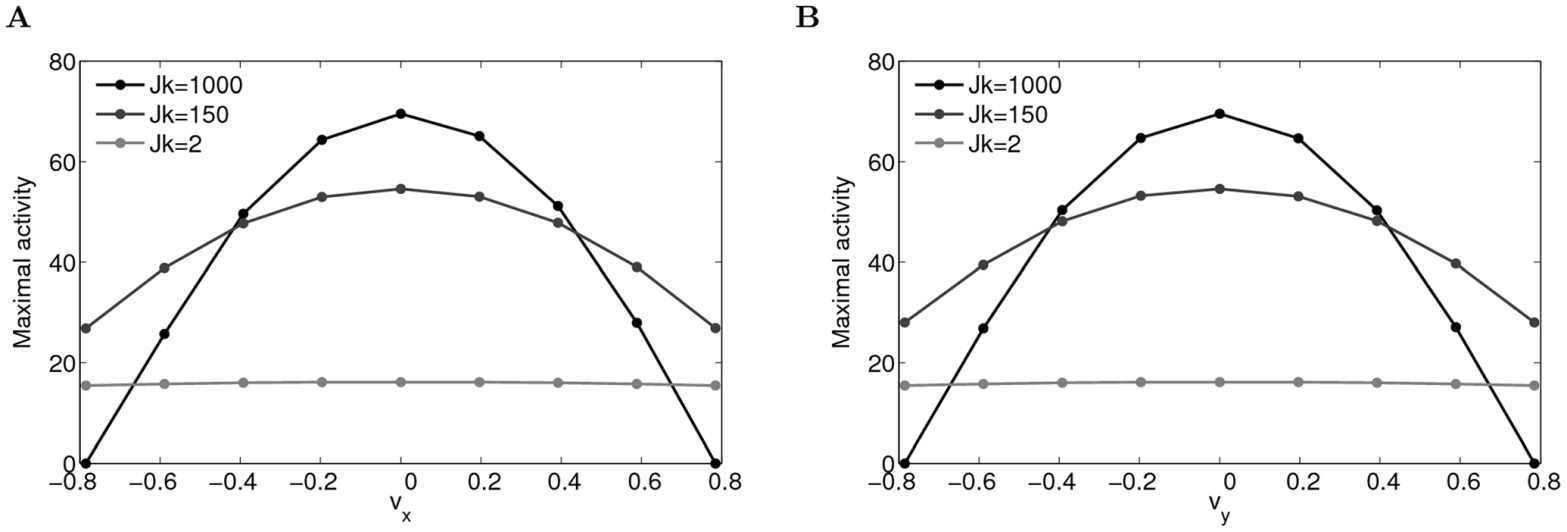
The network activity changes from homogeneous to localized profile in the velocity dimensions with increasing 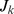 (parameters: 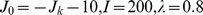). (A) The maximal activity of the units with the same 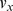 labels for different 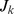; (B) The maximal activity of the units with the same 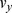 labels for different 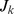. Due to the symmetry in velocity labels, the plots in (A) and (B) are the same.

In two-dimensional environments, the state of the network shows similar transitions as in the case of one dimensional environments. For small 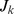, the activity of the network is homogeneous. When 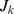 increases, multiple bumps appear forming a triangular lattice in the spatial dimensions, however the activity of the network is not localized in velocity axis, and the network state is static. When 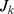 is sufficiently large, the network activity is localized in the velocity axis, and due to the asymmetry in connections, the bumps start to move along the spatial axis. As shown in Fig. 8, the maximal activity of the units with the same velocity label changes from a homogeneous solution (light gray lines) to a localized solution (black lines) as 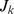 increases.

The input to the network is given by
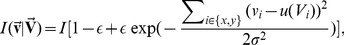
(21)where 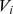 is the component of the velocity vector of the animal in x or y axis of the physical space. 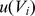, defined in Eq. 16, gives the location of the bumps on the corresponding velocity axis in the neural space. 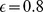 and 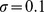 are the strength and the width of velocity tuning.

#### Grid maps in two-dimensional environment

**Figure 9:**
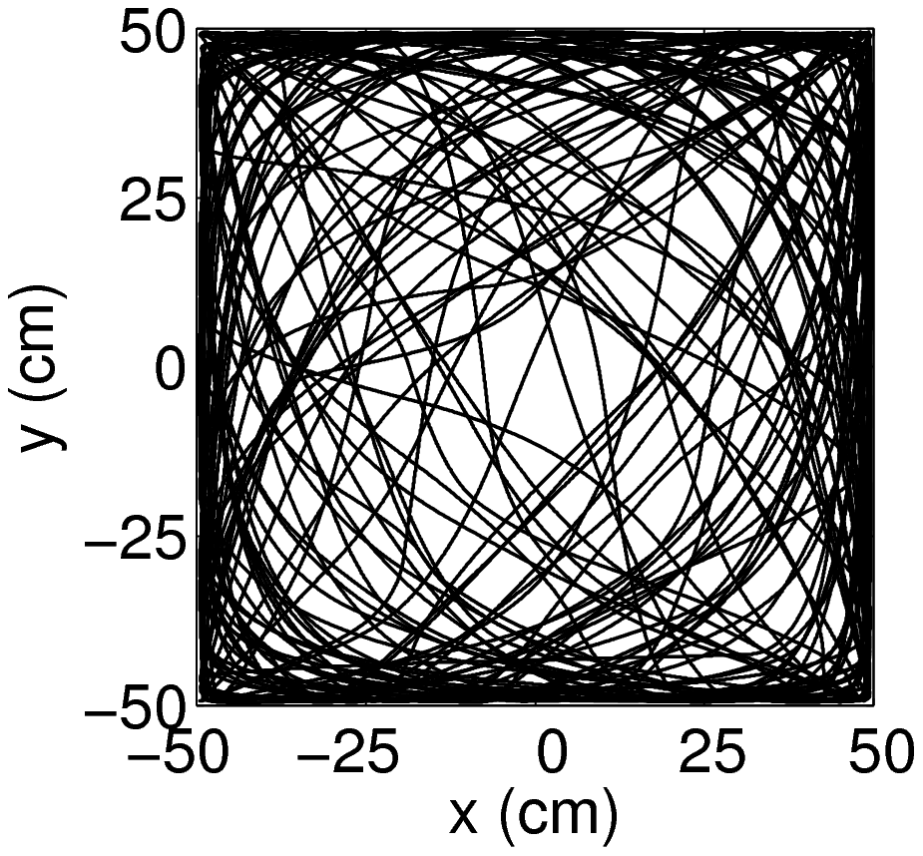
A sample trajectory of the simulated animal in a two-dimensional square environment. The animal is not allowed to move beyond the boundary of the environment. The speed of the animal varies between [0, 100] cm/s.

An animal is simulated to explore a 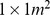 square environment by a smooth random walk (Fig. 9). The velocities in 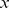 and 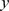 directions vary independently between 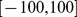 cm/s as in the case of the linear track.

**Figure 10:**
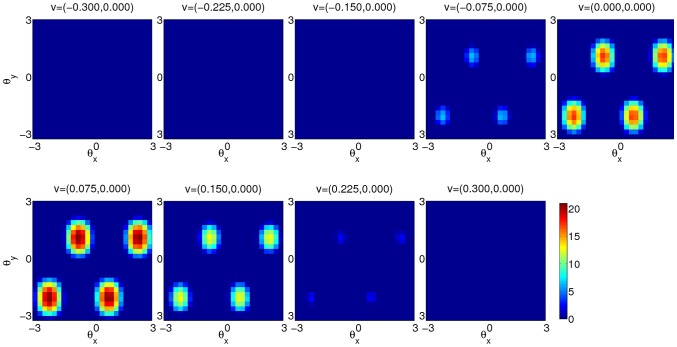
A snapshot of the network activity of the units that prefer zero velocity in y direction (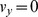) when the animal runs with velocity. 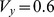
 cm/s and 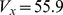 cm/s. Each panel shows the activity of the units on the slice with the fixed velocity labels. The velocity labels of the slice are shown at the top of each panel.

Since it is difficult to visualize the activity in a four-dimensional neural space, Fig. 10 only shows the activity of the units with 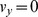. The population activity of the units on each velocity slice has the same triangular lattice structure. In each slice with non-zero activity, the number of bumps is four, because the network accommodates exactly two bumps on each spatial axis. The network activity is centered at the desired position in the velocity dimensions of the neural space and falls off on two sides due to motion-specific input in the Eq. 21.

**Figure 11:**
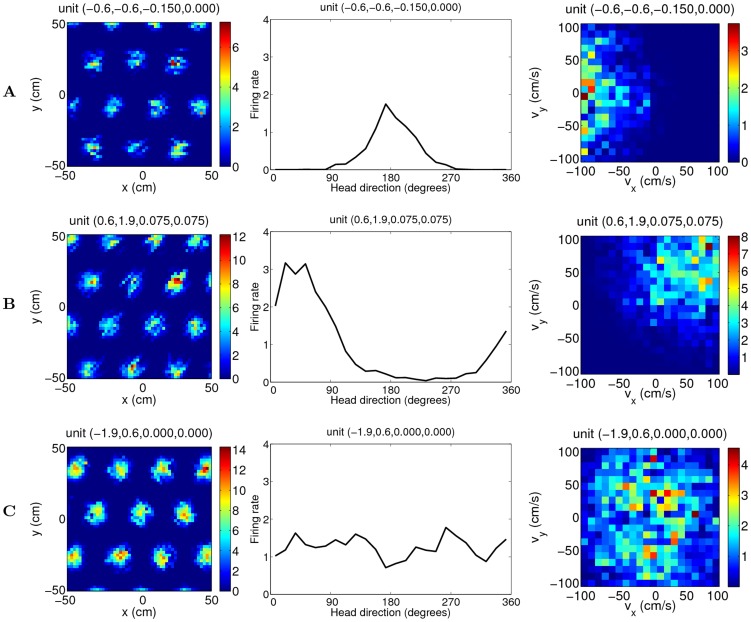
Mean activity of three example units in the network during 20-minute exploration depicted as a function of position (left), head direction (middle) and velocity (right) of the simulated animal (parameters: 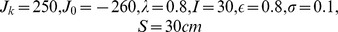). (A,B) Conjunctive units; (C) Grid unit.

The average spatial responses of the units in the network during 20-minute exploration show grid patterns with the same spacing and orientation but variable spatial phases (Fig. 11 left). In addition, the units that are away from the origin of the velocity axes of the neural space show modulation in head directions (Fig. 11A–B middle), similar to the conjunctive cells observed from layer III–VI of MEC [9]. The speed maps of the example conjunctive units verify their preference for fast movements to the west and northeast respectively (Fig. 11A–B right). For comparison, units that are located close to the origin at the velocity axes develop pure positional firing maps (Fig. 11C).

#### Elliptical grid maps

**Figure 12:**
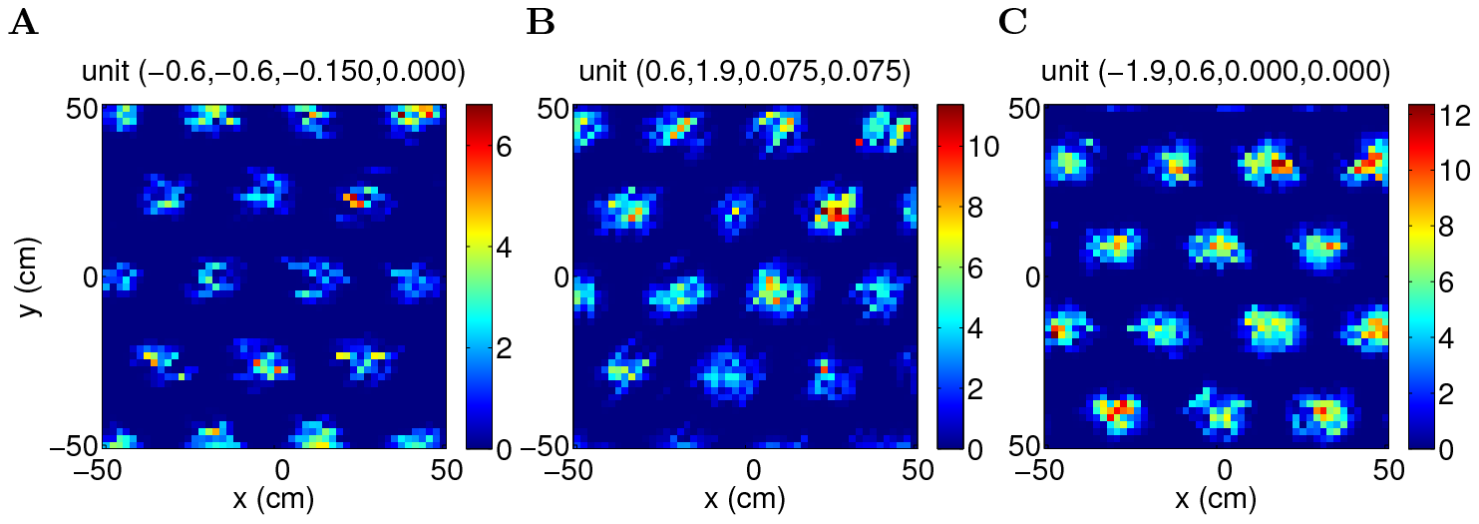
Elliptical grids form if the mapping 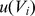 is different for each velocity component (parameters: 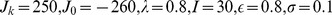). The scaling factor 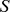 is 30 cm for the mapping in x direction, and is 24 cm for y direction, reduced by 20%. A–C: three different units.

During postnatal development of MEC, the mapping shown in Eq. 21 may not be identical for each velocity component. If the scaling factor in 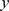 direction is reduced by 20% (S = 24 cm for y direction vs. S = 30 cm for x direction), the activity bumps in the network travel faster in y compared to x dimension. The grid maps formed are compressed by a factor of about 1.2 in the 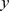 dimension (Fig. 12 vs. Fig. 11 left). The bias in the velocity mapping leads to distorted grids. This might be the underlying mechanism for the observed elliptical arrangement of the surrounding fields instead of perfect circular arrangement seen in an ideal grid [18].

#### Robustness of the network in two dimensional environments

**Figure 13:**
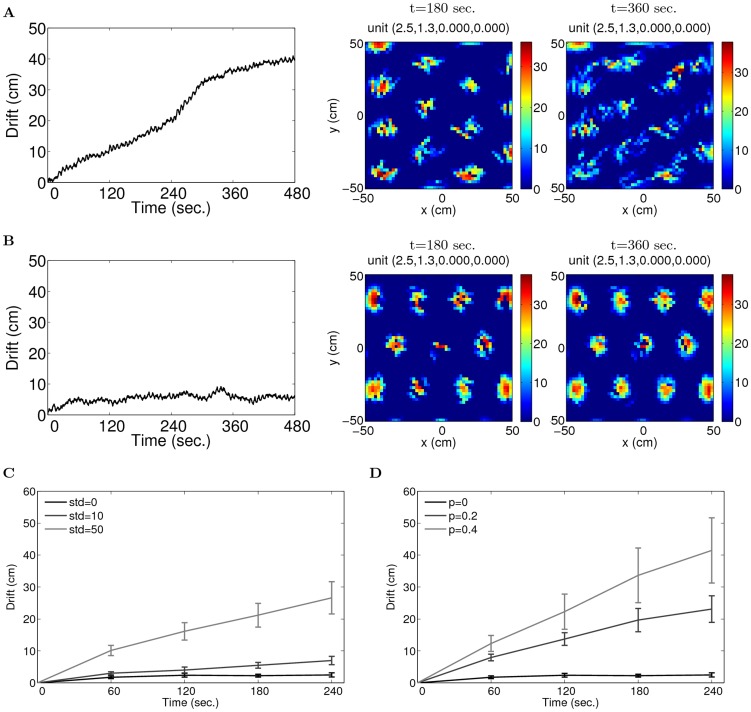
Robustness of path-integration in two dimensional environments when the weights are perturbed by Gaussian random numbers (A) or are deleted randomly (B). Parameters: 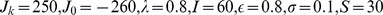 cm. (A) One simulation with the weights perturbed by 10% Gaussian random numbers. Left: drift. middle: fields of a unit in the network after three minutes of exploration. Right: fields of an example unit in the network after six minutes of exploration; (B) One simulation with the weights diluted by 20%. Left: drift. middle: fields of a unit in the network after three minutes of exploration. Right: fields of an example unit in the network after six minutes of exploration; (C) Averaged drift across 8 independent simulations; the network is able to path integrate for 2 minutes (the mean drift within 15 cm, i.e. half of the grid spacing, light gray line) with 10% Gaussian perturbation in the weights, relative to the range of the weights. The black and dark gray lines show the drifts with no and 2% perturbation respectively. Error bars show 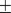 standard deviations; (D) When 20% of the weights are set to zero, the network is able to path-integration for 2 minutes on average (the mean drift across 8 independent simulations kept within half of the grid spacing, dark gray line). The black and light gray lines show the drifts with zero and 40% dilution respectively.

In order to test the robustness of path-integration in two dimensional environments, we perform simulations with random perturbations in the weights. Fig. 13A shows one simulation after adding to the weights of the network random numbers from a Gaussian distribution with zero mean and standard deviation 10% relative the the range of the weights. We reconstruct the position of the animal from the network state and calculate the drift in path integration as the distance between the reconstructed and the actual position of the animal. The drift is kept within half of the grid spacing for three minutes, and the units in the network show grid fields in the environment (Fig. 13A, middle panel). After six minutes, the drift accumulates beyond the grid spacing, and the grid fields start to loose periodic lattice structure (Fig. 13A, right panel). The simulation shown in Fig. 13B is subject to the deletion of 20% of the weights. The drift in path integration is smaller than half grid spacing during the simulation, and grid fields of the units in the network are stable. The network is able to path integrate robustly in two dimensional environments for about 2 minutes with 10% Gaussian perturbation or 20% random dilution in weights (Fig. 13C–D).

## Discussion

In this study, we presented a robust continuous attractor network model to explain the responses of pure grid cells and conjunctive grid-by-head-direction cells in MEC. The main novel assumption of our model is that grid cell system represents different conjunctions of positions and movements of the animal. Neurons in our network occupy a manifold spanned by spatial axis and velocity axis, and are interconnected by asymmetric recurrent connections. Multiple regularly spaced activity bumps localized in all dimensions emerge in the network, and are able to move intrinsically with a range of possible speeds in all directions. The velocity of the bumps depends on their position on the velocity axis. A motion-specific input shifts the bumps along the velocity axis to the corresponding position, so that the velocity of the bumps in the neural space is proportional to the velocity of the animal in the physical space. This linear relation is robust against random perturbations in connections. Thus our model is able to perform robust path-integration. We note that the network model similar to the one corresponding to one-dimensional environment and having one activity bump (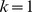, see Eq. 1) could also describe the head-direction system which performs integration of angular velocity.

### Origin of conjunctiveness

Our model accounts for the conjunctive position-by-movement responses of the cells find in deep layers of MEC [9]. This is because the recurrent weights between units are modulated in the velocity axis (Eq. 1), so that in each activity patch only units with similar velocity labels and similar position labels are active. In previous models [3], [5] the incoming weights of the units with the same position labels do not depend on their velocity tuning, therefore they must be active together. Units may gain weak degree of conjunctiveness by scaling up the amplitude of velocity input, so that units that are not driven by strong velocity input will be less active. But since this is not a stable attractor state of the network, strong conjunctiveness will push the network out of the stable regime.

In rodent MEC, pure grid cells and conjunctive cells coexist in the same module [18]. Conjunctive cells exist in layer III, V and VI. Pure grid cells are found in layer II, and are mixed with conjunctive cells in deep layers [19]. Overall, the proportion of conjunctive cells among all grid cells is no more than 50%. In our model, the conjunctiveness of a unit is correlated with its absolute velocity label. Grid cells have velocity labels close to the origin (closer than half the size of the bump), hence they are active for all movement directions. Cells that are further away from origin in the velocity axis are only active when the animal moves in a particular direction, thus resulting in head-direction selectivity in addition to position response as pure grid cells. The ratio between the number of pure grid units and the number of conjunctive units depends on the size of the bumps: the larger the size of the bumps, the larger the number of pure grid cells.

### Velocity input

The model requires precise velocity input indicating the direction and speed of animal movement. MEC may receive velocity-tuned input from posterior parietal cortex and retrosplenial cortex [20]–[24]. These regions integrate multimodal sensory information, such as movement information from vestibular system relayed by thalamus and optical flow information from visual cortex, and play an important role in spatial navigation [25]–[28]. In rodents, many of the cells in posterior parietal cortex have been found to respond to velocity and acceleration [29]. Therefore, posterior parietal cortex can be one possible source of self-motion signal for MEC network [30].

The connections from movement-selective cells in posterior parietal cortex to MEC cells can be tuned during postnatal development, and map animal movement to the position of the activity bumps on the velocity axis (Eqs. 16 and 21). The possibility to precisely learn such a mapping allows for the flexibility in MEC intrinsic connectivity and neural firing mechanisms. The coupling between units is not necessarily restricted to a cosine shape, as analyzed here. The firing rate of each unit can depend nonlinearly on its input, e.g. as a sigmoid transfer function.

The parameter 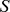 that defines the ratio between the flow of the activity pattern and the velocity of the animal (see Eq. 15) determines the grid scale of a MEC module. From dorsal to ventral, MEC units are arranged into local modules with increasing discrete values of 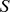, resulting in discretized grid scales [18]. If there is a bias in the connectivity, e.g. movement-selective cells are systematically connected to MEC cells with larger absolute velocity labels along one velocity axis of the neuronal space, MEC cells will express elliptical grids (Fig. 12), as observed experimentally [18].

### Different modes of navigation

Accumulating experimental evidence shows that mammals adopt two types of navigation. Path-integration is useful when landmarks are not available, e.g. in the darkness or when a cognitive map representation is being learned after entering in a novel environment. Map-based navigation is able to reset the error in path-integration, calibrating the internal spatial representation according to the external landmarks. The dynamics of the spatial representation in the brain depend on the interaction between these two modes of navigation. Integration of these two modes in a network model may better explain the responses of grid cells in novel environments or after environment changes [31].

### Predictions of the model

Several testable predictions can be derived from the model. To verify these predictions, new experiments and analysis should be carried out to examine the selectivity of the responses of MEC principle cells.

#### Gradient of head-direction selectivity

The assumption of the intrinsic representations of conjunctions of positions and movements of the animal would predict that MEC principle cells show continuous gradient of head-direction selectivity. Cells with strong head-direction selectivity would fire preferentially at fast speed, and cells with weak head-direction selectivity would prefer low speed. There would be substantial amount of grid cells showing intermediate head-direction selectivity, firing maximally at an intermediate speed.

#### Conjunctive cells have lower firing rates compared to grid cells

**Figure 14:**
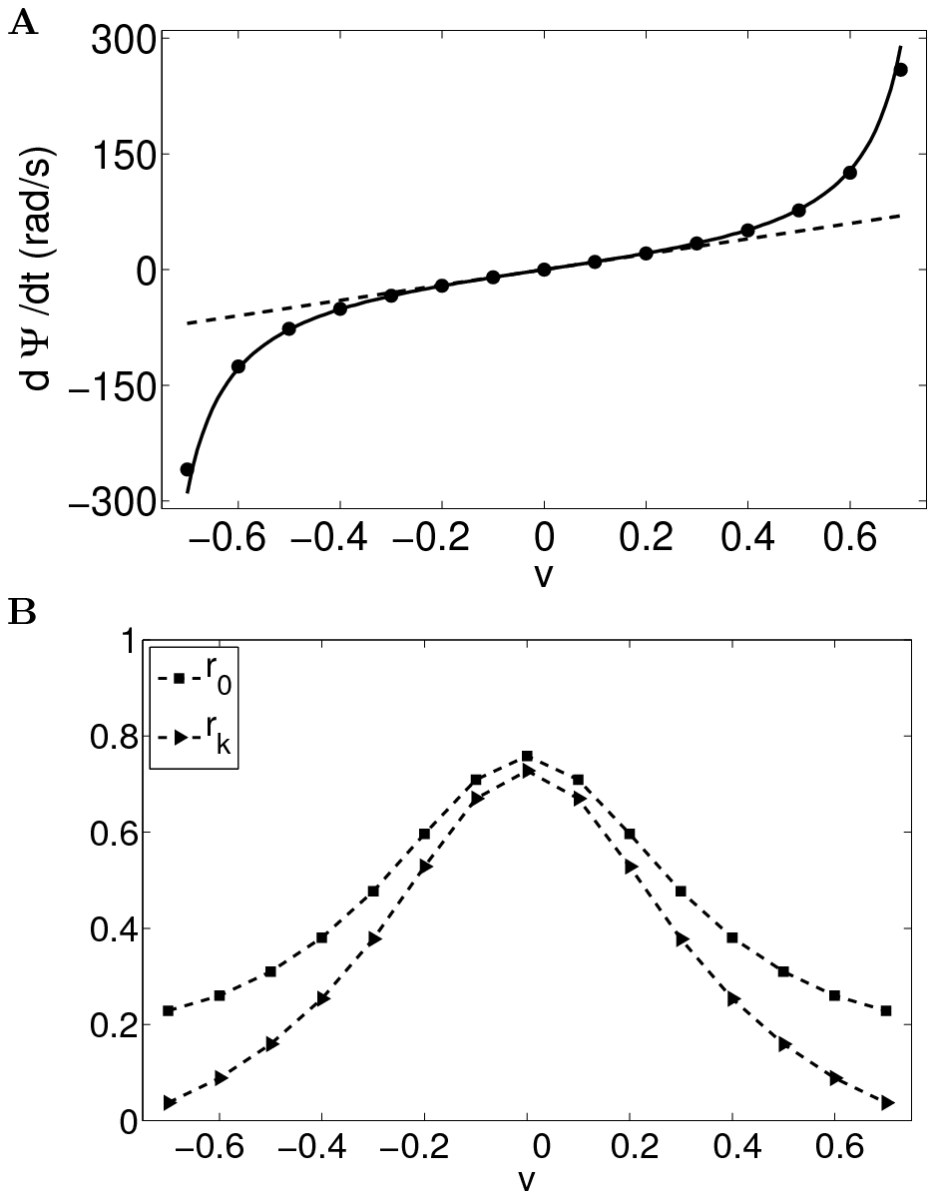
Estimated order parameters of the traveling multiple bumps. (A) Estimated velocity of the bumps (filled circles) for different 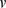 matches the theoretical values (solid line, Eq. 55). The linear approximation of the velocity of the bumps is plotted as the dashed line; (B) When the absolute value of 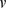 goes to the limit, the network has homogeneous activity, with finite mean activity 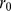 (square markers) and vanishing amplitude of the bumps 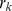 (triangular markers).

Both the peak activity and the mean activity of the network are smaller when the bumps moves with faster intrinsic velocity (Fig. 14B). This leads to the prediction that, on short time scale, the peak firing rate of a conjunctive cell along its preferred head direction would be lower than the peak firing rate of a grid cell. On long time scale, the multiple place fields of a conjunctive cell in an environment would have lower peak activity along its preferred firing direction than those of pure grid cells.

#### Traveling waves in the absence of self-motion input

In the model, when movement-specific input is absent (i.e. uniform input), the bumps are free to stabilize at arbitrary positions on the velocity axis of the neural space. Afferent input from posterior parietal cortex and vestibular system has been shown to be important for path-integration [32], [33]. In animals with damaged connections from posterior parietal cortex to MEC, or bilateral vestibular deafferentation, spontaneous traveling waves would appear in MEC. It could be possible to record sequences of bursting activity in MEC cells from these animals when they are stationary.

#### Shift of spatial fields in running directions

In the simulations, the position-by-velocity fields expressed by a unit are slanted in the spatial dimension (Fig. 4E and 6B). The peak position of the spatial fields in different velocity ranges shows a shift toward running directions. The shift results from asymmetric connections between MEC units. As a prediction, the spatial fields of a grid cell when the animal runs along one direction would be offset slightly toward the running direction as compared with the spatial fields of the opposite direction.

#### Synaptic plasticity in the projections from posterior parietal cortex to MEC

In the model, the mapping between movement-sensitive units and MEC units should be setup during some learning phase. The projections from regions like posterior parietal cortex to MEC may function as such a mapping. These projections are likely to mature in postnatal day 16 to 25, during which grid cells develop periodic grid firing pattern [14]–[16]. Two predictions can be made about the interaction between MEC and posterior parietal cortex. First, during appropriate developmental stage, strong synaptic plasticity in these projections should be observed in juvenile animals as compared to adult rats. Second, the synchrony between the cells in posterior parietal cortex and MEC cells should increase during development, because the information flow between these two regions becomes more evident when the connections between them become stronger and more accurate.

## Methods

### Order parameters

The network activity can be characterized by a set of order parameters derived from its Fourier transform
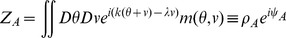


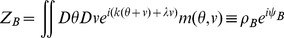
(22)

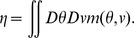



The dynamics of the network activity can be written as
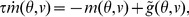
(23)where 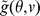 is the total input to a unit given by
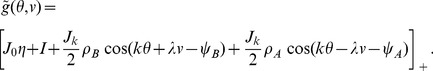
(24)


Fourier transforming the firing rate dynamics Eq. 23 reveals the dynamics of the order parameters 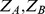, and 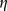


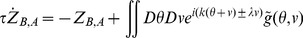
(25)

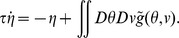



The solutions of the dynamics can be better described by recombining the order parameters in Eqs. 22 into the following dimensionless quantities
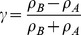


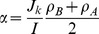


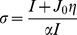
(26)

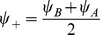


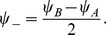
With this set of order parameters and by defining the rescaled gain function
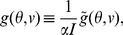
(27)
Eq. 23 results in Eq. 4.

Differentiating Eqs. 26 and combining Eqs. 25 lead to the dynamics for the reduced order parameters in Eqs. 6.

Due to the asymmetry of the coupling in 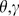 is not zero. This can be seen by linear expansion of the integrand at 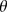 in the first Eq. of 6 and assuming 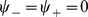.
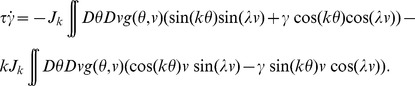
(28)

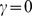
 does not satisfy the second term of the right hand side of the above equation, since 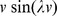 is an even function.

### Stability of a homogeneous solution

In the homogeneous regime, the order parameter 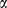 vanishes at the steady state. We introduce a new order parameter 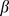, being the size of the bumps
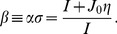
(29)

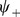
 and 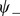 are two free parameters. We choose them to be 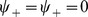. It is sufficient to consider the dynamics of 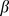 and 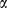. By using the derivative chain rule, the dynamics of 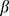 and 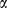 can be obtained from Eq. 29 and Eqs. 6,
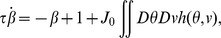


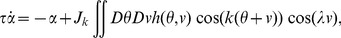
(30)where 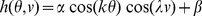.

The stability of the homogeneous solution can be inspected by linearizing Eqs. 30 at the fixed point 
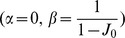
. The matrix governing the linear dynamics of perturbations reads
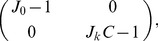
(31)where 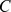 is given in Eq. 9. Therefore, the conditions for the homogeneous solution to be stable are 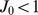 and 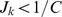, as shown in Eqs. 7–8.

### Onset of traveling bumps

We analyze the onset of the freedom of choice of 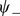. In this case, the bumps just touch the boundaries of the 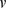 range. Posing 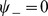, and the steady state activity at at 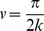 is
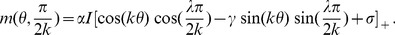
(32)The angle 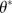 at which this activity is maximal is
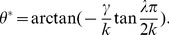
(33)


So the maximal activity at the 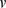 boundaries is
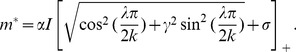
(34)


For 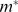 to vanish,
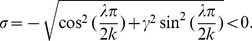
(35)


### Network simulations

The time constant 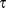 is set to 10 ms throughout the paper. Differential equations are numerically integrated according to the fourth order Runge-Kutta method. The time-step for numerical integration is 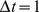 ms.

For the path-integration simulations on linear tracks, the virtual animal runs back and forth, and changes its running direction only at the two ends of the track. The velocity of the animal is determined according to
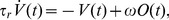
(36)where 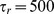 ms is the time constant. 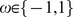 is the current running direction. 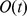 is a piecewise constant function, whose values are sampled uniformly in [0,100] cm/s every second. When the animal is approaching close enough to the end of the track (distance to the end of the track smaller than 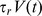), O(t) is set to zero to make sure that the speed of the animal decreases to zero at the end of the track. In this deceleration phase, once the speed of the animal drops below 5 cm/s, the animal reverses its direction 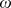 and a new value of 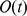 is chosen randomly. The trajectory in a 2D environment is simulated by two such independent random walkers.

For a spacing 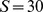 cm, velocity 100 cm/s in physical space would require the bumps centered at 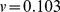 on the velocity axis. In the path-integration simulations, we reduce the range of the velocity label 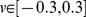, which is enough to cover the velocities experienced by the rat.

The neural space is discretized into equal-sized bins, with each bin occupied by one unit. In the model for linear track, the 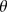 axis is divided into 200 bins and the 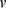 axis into 51 bins. The network is composed of 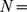
10,200 units. In the model for two-dimensional environments, the axes 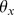 and 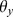 are divided into 25 bins each and the axes 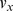 and 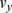 into 9 bins each, resulting in 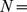
50,625 units in total.

### Estimating the intrinsic velocity of the bumps

Initially the network is given stronger input for units with specified 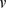 coordinate to let the network form bumps at the corresponding position of the 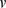 axis. The input is gradually decreased to uniform. The network is simulated for one second with uniform input, and the center of the bumps on 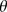 axis and 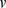 axis are estimated at each time step by
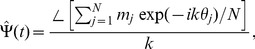
(37)

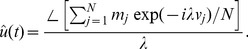
(38)where 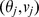 is the coordinate of unit 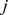 in the neural space. 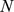 is the total number of units in the network. 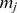 is the activity of unit 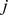. Function 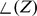 takes the angle of a complex number 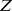.

The velocity of the bumps is calculated as
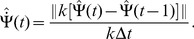
(39)Here 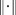, defined in Eq. 20, measures the distance considering periodic boundary condition.

### Speed estimation in the asymmetric ring model

Here we consider a simplified case in which the manifold 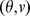 is reduced to a ring on 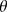. On the ring, the units have different labels in 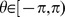 but the same label of 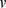.

The interaction between two units 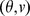 and 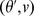 is
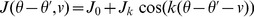
(40)where 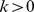 is an integer.

The rate dynamics are defined by
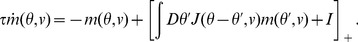
(41)


The state of the system can be described in the frequency domain as in the Fourier transform of the activity 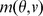 reads
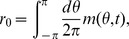


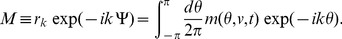
(42)The mean activity 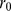, the magnitude 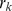 and the phase 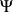 of the 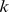-th component of the Fourier transform are defined as the order parameters of the system. The dynamics in Eq. 41 can be rewritten into a simpler form in terms of the order parameters
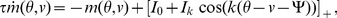
(43)where 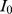 and 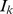 are given by
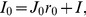
(44)

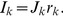
(45)


Fourier transforming Eq. 43 results in the dynamics governing the order parameters
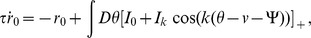


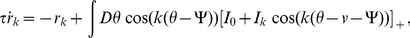
(46)

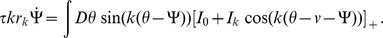



#### Narrow activity profile

For narrow activity, there exists 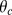


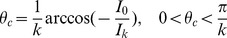
(47)such that Eqs. 46 can be wrriten as
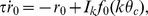


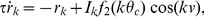
(48)

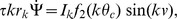
where 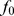 and 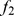 are given by
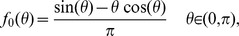


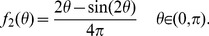
(49)In the range 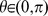, 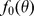 and 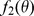 are monotonic functions. 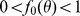 and 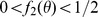.

From Eq. 48, the steady states of order parameters 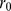 and 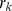 are
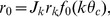
(50)

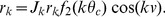
(51)


Imposing 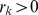 in Eq. 51 leads to the condition for localized solution of 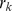


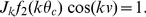
(52)Considering the upper bound on the function 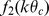, Eq. 52 implies a limited range of 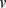


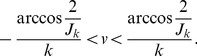
(53)When 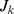 goes to infinity, this condition is simply
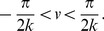
(54)


The velocity of the bumps can be derived from Eq. 48 and 52

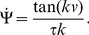
(55)


From Eq. 50 and 51

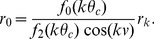
(56)


Putting Eq. 56, 44 and 45 into Eq. 47, the amplitude of the bump 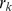 is given by (see Fig. 14B for numerical results)
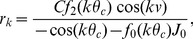
(57)which leads to the condition from amplitude instability
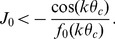
(58)



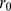
 is given by
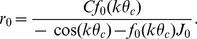
(59)


Finally, putting Eq. 59 and 57 into Eq. 44 and 45, 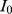 and 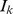 are
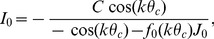
(60)

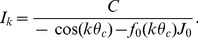
(61)


#### Speed of traveling bumps

Eq. 41 is simulated to estimate the speed of traveling bumps. 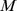 is estimated by
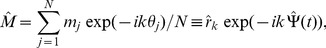
(62)where 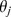 is the position label of unit 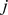 and 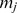 is the corresponding activity. 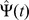 and 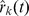 are determined from 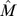. The velocity of the bumps is estimated according to Eq. 39. Fig. 14A shows that the estimated velocity matches the analytical result in Eq. 55. The bumps exist as long as 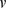 does not go to the limit (Fig. 14B), in which the amplitude of the bump vanishes.

## Supporting Information

Video S1 **Path-integration in a 1D environment.** Top-left panel: virtual animal running back and forth on a two-meter-long linear track. Top-right panel: time evolution of network activity. Bottom-left panel: Position of the animal on the track (red - actual position; black - estimated position). Bottom-right panel: velocity of the bumps vs. velocity of the animal.
(AVI)
